# Photochemical Modification of the Extracellular Matrix to Alter the Vascular Remodeling Process

**DOI:** 10.3390/jfb14120566

**Published:** 2023-12-15

**Authors:** Blake Anderson, Dylan Blair, Kenji Huff, John Wisniewski, Kevin S. Warner, Katalin Kauser

**Affiliations:** 1 Biology Department, Alucent Biomedical Inc., Salt Lake City, UT 84108, USA; banderson@alucentbiomedical.com; 2 Engineering Department, Alucent Biomedical Inc., Salt Lake City, UT 84108, USA; dblair@alucentbiomedical.com (D.B.); khuff@alucentbiomedical.com (K.H.); 3 Pharmaceutical Development, Alucent Biomedical Inc., Salt Lake City, UT 84108, USA; jwisniewski@alucentbiomedical.com (J.W.); kwarner@alucentbiomedical.com (K.S.W.)

**Keywords:** vascular disease, photosensitized protein crosslinking, extracellular matrix, bio-scaffold, tissue remodeling, vascular remodeling, natural vascular scaffolding

## Abstract

Therapeutic interventions for vascular diseases aim at achieving long-term patency by controlling vascular remodeling. The extracellular matrix (ECM) of the vessel wall plays a crucial role in regulating this process. This study introduces a novel photochemical treatment known as Natural Vascular Scaffolding, utilizing a 4-amino substituted 1,8-naphthimide (10-8-10 Dimer) and 450 nm light. This treatment induces structural changes in the ECM by forming covalent bonds between amino acids in ECM fibers without harming vascular cell survival, as evidenced by our results. To further investigate the mechanism of this treatment, porcine carotid artery segments were exposed to 10-8-10 Dimer and light activation. Subsequent experiments subjected these segments to enzymatic degradation through elastase or collagenase treatment and were analyzed using digital image analysis software (MIPAR) after histological processing. The results demonstrated significant preservation of collagen and elastin structures in the photochemically treated vascular wall, compared to controls. This suggests that photochemical treatment can effectively modulate vascular remodeling by enhancing the resistance of the ECM scaffold to degradation. This approach shows promise in scenarios where vascular segments experience significant hemodynamic fluctuations as it reinforces vascular wall integrity and preserves lumen patency. This can be valuable in treating veins prior to fistula creation and grafting or managing arterial aneurysm expansion.

## 1. Introduction

The extracellular matrix (ECM) stands as a fundamental constituent in living organisms, comprising a three-dimensional framework of structural and functional proteins involving nearly 300 member proteins [1]. Throughout development, it plays a pivotal role in shaping tissues and maintaining the functional equilibrium in mature tissues. Additionally, the ECM continually adjusts in response to environmental signals, conveying biochemical or biomechanical cues to the cellular elements within the tissue [2]. The control of cellular functions by the ECM heavily relies on integrin signaling, forming the basis for tissue remodeling in both health and disease [2].

The ability for adaptive remodeling is particularly crucial for tissues of the cardiovascular system, including the heart, arteries, and veins. The constant hemodynamic fluctuations stemming from normal physiological cardiovascular functions necessitate enhanced adaptability in both cellular and ECM constituents within the vascular wall, compared to more static organs. Conditions such as atherosclerosis and hypertension subject the vessel wall to repetitive biochemical and mechanical stresses due to inflammation and elevated blood pressure, consequently initiating excessive vascular remodeling. Maladaptive remodeling often underlies the development of severe cardiovascular complications, particularly as the aging ECM struggles to maintain a homeostatic balance owing to its diminishing mechanical properties [3]. This can lead to thickening of the vessel wall with lumen constriction, as seen in peripheral arterial disease, or thinning of the vessel wall in the case of aneurysms [4]. These alterations exemplify maladaptive vessel wall remodeling, where ECM changes result in unregulated cellular behavior, leading to the shift of contractile vascular smooth muscle toward a proliferative or secretory phenotype, or triggering cell death [5,6].

Vascular wall remodeling begins as an adaptive mechanism with the primary aim of preserving adequate organ perfusion. Therefore, the mechanosensation of blood flow through appropriate cell–ECM interactions plays a pivotal role in governing this physiological response [3,5]. The establishment of vascular access for hemodialysis in patients with end-stage kidney disease (ESKD) capitalizes on this natural adaptive process. Arteriovenous fistula (AVF) creation introduces a new hemodynamic environment to the veins. The successful maturation of a fistula, known as arterialization, hinges on a carefully orchestrated tissue adaptive response, which leads to outward remodeling [7]. The quality and arrangement of the ECM, which governs cellular responses that drive outward-directed proliferation, ensure the requisite thickening of the vein wall without compromising the patency of the venous lumen [8,9,10].

The significance of the ECM’s quality and integrity in regulating vascular remodeling processes is gaining increasing attention as our understanding of its role in governing vascular adaptation responses expands [4,10]. Substantial efforts have been invested in developing engineered ECM scaffolds that mimic the natural mechanical and biological characteristics of the ECM [11]. Alternatively, methods are being developed to produce natural ECM scaffolds using cellular reactors, creating bioengineered conduits for vascular applications [12]. Targeting the ECM to combat vascular disease has also gained traction through pharmacological approaches [3]. These approaches primarily focus on enzymatic processes contributing to ECM remodeling or the functional aspects of cell–ECM interaction. A more direct alteration of the ECM involves modifying the degree of crosslinking between the structural proteins that compose the matrix. Various methods have been devised for this purpose, ranging from chemical crosslinkers to photooxidative techniques. Chemical crosslinkers like glutaraldehyde, used to stabilize ECM scaffolds primarily consisting of collagen, can lead to highly crosslinked scaffolds that often provoke inflammatory responses and foreign body reactions, limiting their utility [13]. In contrast, photochemical methods utilizing photosensitizing agents and targeted light activation within the ECM establish a more natural linkage pattern, creating resistance to enzymatic protein degradation without toxicity and compromised biocompatibility [14].

A vascular device combined with the Natural Vascular Scaffolding technology has been developed to optimize ECM modification, facilitating adaptive tissue remodeling for vascular applications. This innovative technology employs a 4-amino substituted 1,8 naphthalimide (10-8-10 Dimer), which has an absorption maximum at 450 nm. This photosensitizer initiates a photochemical reaction, resulting in protein crosslinking by establishing covalent bonds among oxidizable amino acids within the vascular ECM’s protein structure [15,16]. Implementing the photochemical treatment at the time when the blood vessel segments are stretched by an intravascular balloon or arterial blood flow ensures that the newly formed crosslinks maintain the expanded patent lumen size, serving as a flexible scaffold. The ECM’s stretch and rearrangement trigger the adaptive cellular response, with proliferation remaining directional due to the reconnected matrix proteins and reduced inflammatory response [9,17].

This paper illustrates the dose-dependent covalent bond formation through the photochemical reaction and demonstrates the preserved cellular functionality of the treatment while significantly enhancing the ECM scaffold’s resistance to enzymatic degradation, compared to segments treated with plain old balloon angioplasty (POBA) (i.e., balloon inflation of blood vessel segments without the photochemical treatment). These findings offer a mechanistic explanation for previously observed in vivo effects of the treatment [9,17] and contribute to the advancement of this technology toward clinical development.

## 2. Materials and Methods

### 2.1. Photochemical Dimerization of Tyr-Containing Pentapeptides

The 10-8-10 Dimer was synthesized by Syngene Intl (Bangalore, India) and pentapeptide Ac-AKGYG-NH2 was synthesized in Dr. Andrew Roberts lab at the University of Utah. An Alucent Light Fiber coupled Laser System (LaserGlow, Toronto, ON, Canada) fitted with a frontal diffuser (Medlight, Ecublens, Switzerland) was used to supply the 450 nm light. A Field Max II Detector with PM10 Sensor (Coherent, Santa Clara, CA, USA) was used to determine the distance to place the frontal diffuser away from the reaction solution. The distance was chosen such that the desired light dose was delivered in a 2 min time period for each condition.

The 10-8-10 Dimer was weighed out and then dissolved in water to a concentration of 125, 100, 30, and 5 mg/mL. The pentapeptide Ac-AKGYG-NH2 was weighed out and dissolved in water to a concentration of 65 mg/mL. The amount of peptide used was based on the molarity of collagen present in veins. The 10-8-10 Dimer concentrations were chosen as therapeutically relevant values based on prior drug delivery studies [17]. A 10-8-10 Dimer concentration of 2500 µg/mL in solution is equal to a tissue concentration of 2500 ng/mg (1 mL of water = 1000 mg of tissue by weight). To a 20 mL scintillation vial, 950–980 µL of 100 mM sodium phosphate buffer was added, and then 10 µL of the pentapeptide stock solution, followed by 10–40 µL of the appropriate 10-8-10 Dimer stock solution. The resulting mixture was allowed to stir for 1 min with no light, and then the light source was switched on and the solution was exposed to 450 nm light at the preadjusted distance for 2 min to achieve light dose exposure. After light exposure, the light source was turned off and an aliquot was taken and the amount of dimerized pentapeptide was analyzed via HPLC (Agilent Technologies, Inc, Santa Clara, CA, USA) using a Waters XTerra MS C18 Column (Waters Corporation, Milford, CT, USA) 125 Å, 5 µm, 4.6 mm × 250 mm. The mobile phase consisted of the following: 98.9% water, 1% acetonitrile, 0.1% TFA (mobile phase A) and 89.93% acetonitrile, 10% water, 0.07% TFA (mobile phase B). The gradient used was 0% B for 1-min, linear gradient 0% to 40% B over minutes 1 to 12, isocratic at 40% B from 12 to 15 min, and then isocratic at 0% B from 15 to 20 min. A 10 µL injection volume, 1 mL/min flow rate, and detection at 254 nm were also employed.

### 2.2. Cell Culture and Evaluation of Cell Viability following Photochemical Treatment

Cryopreserved human umbilical vein endothelial cells (HUVEC) were obtained from PromoCell (Heidelberg, Germany), thawed, and plated in cell culture flasks at a density of 2.5 × 10^3^ cells/cm^2^ in phenol red-free Endothelial Cell Growth Medium (PromoCell, Heidelberg, Germany) supplemented with Growth Medium Supplement Mix (PromoCell, Heidelberg, Germany). Cells were subcultured from the flasks and were plated at 5000 cells/well in 96-well plates and incubated for ~24 h prior to treatment exposure including the 4 different timepoints (4–72 h) for viability assessments. For treatment, the growth media was aspirated, and the cells were washed once with 200 μL Hanks Balanced Salt Solution (HBSS; SIGMA, St. Louis, MO, USA) with gentle agitation. The HBSS was aspirated and 100 μL of the prepared dosing solutions was added to the appropriate wells. The plates were placed back in the humidified incubator at 37 °C with 5% CO_2_. Shortly after application of the 10-8-10 Dimer solution and controls, one set of identical plates were removed from the incubator and exposed to 450 nm of light using a custom-made light source or kept outside the incubator in the dark for the same amount of time. All drug exposures and untreated controls were tested in quadruplicate (n = 4).

Five concentrations of the 10-8-10 Dimer (12.5, 25, 50, 100, and 200 μg/mL, respectively) were tested. This concentration of 10-8-10 Dimer is 60-1000-fold above the expected concentrations delivered in vivo or during the simulated in vitro treatment conditions, given only one monolayer of cell population representing the tissue mass compared to a multilayered vascular wall. Chlorpromazine (5 μg/mL, 10 μg/mL, and 15 μg/mL) was used as a positive control to assess potential cellular damage due to phototoxicity. The negative control was phenol red-free culture media with 1% final concentration of HBSS. The light-treated plates were an identical set of plates, as it comes to compound treatment, but exposed to 450 nm of light for a 50 s exposure, totaling a light dose of 7 Joules per square centimeter (J/cm^2^), a 6 min 49 s exposure totaling a light dose of 14 J/cm^2^, and a 13 min 38 s exposure totaling light dose of 28 J/cm^2^ light dose, respectively. Two hours after exposure to the drug/light treatment, media was removed from all plates and fresh phenol red-free growth media was added to all plates for the additional time points (4, 24, 48, and 72 h exposure).

At the conclusion of the scheduled time points following 10-8-10 Dimer and light exposure, the HUVEC cells were assessed for viability using 3-(4,5-dimethylthiazol-2-yl)-2,5-diphenyltetrazolium bromide (MTT; Sigma-Aldrich, St. Louis, MO, USA). First, the phenol red-free culture media was removed and 100 μL of room temperature HBSS was added to the wells and then gently aspirated. Then, 100 μL of prepared MTT media (1 mg/mL MTT in phenol red-free culture media) was added to each well. The plates were then incubated for approximately 3 h in a humid atmosphere containing ~5% CO_2_ in the dark at 37.0 ± 2.0 °C. The MTT medium was then removed, and cells were lysed by adding 100 μL isopropanol (Sigma, St. Louis, MO, USA) to each well. Plates were gently shaken for ~20 min on an orbital plate shaker at room temperature. After shaking, the absorption was measured at 570 nm with the BioTek Cytation 5 Microplate Reader (Winooski, VT, USA). Cell viability was calculated for each exposure group as a percentage of the mean absorption value of the negative control wells with untreated cells and after subtraction of background.

### 2.3. In Vitro Preparation and Treatment of Vascular Segments

Carotid arteries from 6–9-month-old swine were obtained from Animal Technologies (Tyler, TX, USA). The arteries were cleaned of loose adventitia. The vascular lumen was gently flushed with phosphate-buffered solution (PBS). The cleaned arteries were then cut into four segments approximately 1.5 cm long. These four segments were then cut in half and were selected for the photochemical (10-8-10 Dimer-treated) and POBA control (POBA CTRL) treatment, respectively. This was performed to account for the heterogeneity between samples selected for photochemical versus POBA treatments.

For the photochemical treatment, samples were soaked in 20 mL of 2 mg/mL 10-8-10 Dimer for 5 min inside a 50 mL conical tube. The concentration of the 10-8-10 Dimer in the soaking solution was selected based on previous experiments [17]. The 10-8-10 Dimer-treated artery samples were removed from the conical tube and rinsed in PBS for 30 s. Following the PBS rinse, a 6 mm diameter × 60 mm length balloon was placed into the 10-8-10 Dimer-treated or control artery and inflated to nominal pressure (6 ATM) using an indeflator (Abbott Vascular, Santa Clara, CA, USA). The inflation was used to provide an approximately 30% stretch above the resting diameter of the arteries. Before this angioplasty step, a light fiber (Alucent Biomedical, Salt Lake City, UT, USA) was inserted inside the balloons, which was connected to a light source (Alucent Biomedical, Salt Lake City, UT, USA). The treatment was performed in two steps. After a 1 min duration of the balloon inflation inside the artery segments, the light source was turned on for another minute for the light fiber to emit a 450 nm wavelength blue light inside the balloon-inflated arterial segment to activate the 10-8-10 Dimer in the vessel wall. The POBA control treatment was performed similarly including the light exposure step, but instead of soaking them in 10-8-10 Dimer, the vessels were soaked in PBS.

Following the photochemical treatment, small segments were snap frozen in liquid nitrogen and stored at −80 °C for subsequent analysis of the 10-8-10 Dimer concentration in the tissue via extraction and HPLC analysis. The frozen samples were thawed, cut to obtain a planar segment, blotted dry on a Kimwipe, and weighed. The weighed segment was placed in a 20 mL glass scintillation vial and for each 100 mg of artery, 3 mL of extraction solution (50:50 acetonitrile:1% TFA in water, *v*:*v*) was added. The samples were incubated on a shaker at room temperature for 3 h and filtered into an HPLC vial. The amount of 10-8-10 Dimer present was determined by HPLC analysis, using an Acquity UPLC HSS PFP 1.8 µm 3 × 100 mm column (Waters Corporation, Milford, CT, USA). The above-described 10-8-10 Dimer application protocol resulted in an average drug mass to tissue mass of 1427 ± 330 ng/mg in the isolated artery sections, which was within the range of 10-8-10 Dimer concentration used for the pentapeptide study.

### 2.4. Elastase and Collagenase Enzyme Exposure

**Figure 1:**
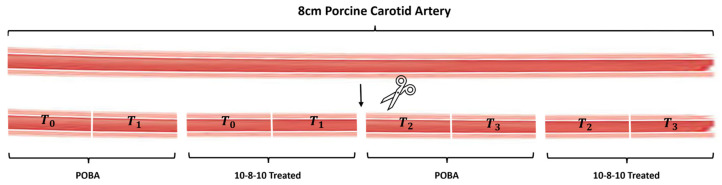
Depiction of how artery segments were assigned to 10-8-10 Dimer treatment (10-8-10 Treated) or POBA control (POBA) and subsequent enzyme exposures.

Approximately 0.5 cm long sections were cut from n = 6 POBA control and 10-8-10 Dimer-treated samples, respectively, following the light treatment (Figure 1). These short segments were then exposed to either 2 mL of 0.5% elastase (ThermoFisher, Waltham, MA, USA) or to 1.5 mL of 10% collagenase-III (Rockland Antibodies and assays, Limerick, PA, USA) in a 2 mL Eppendorf tube. The samples were incubated at 37 °C on a rotator plate for different lengths of times. T0 samples were not exposed to enzyme treatment. T1, T2, and T3 samples were subjected to 1, 2, and 4 h of enzyme treatment, respectively (Figure 1). 

### 2.5. Histology and Image Analysis

The artery samples were removed from the enzyme solutions, rinsed in water, and placed into 15 mL of 10% neutral-buffered formalin (NBF) for enzyme deactivation and tissue fixation overnight after each incubation period. Following fixation, the artery sections were subjected to standard FFPE (formalin fixed paraffin embedded) tissue processing. Blocks were trimmed to minimize histological “edge effect” artifact and then sectioned at 5 µm thickness. Sections were stained for elastin using Elastin Verhoff van Gieson (EVG) stain and Masson Trichrome stain was used for collagen visualization. Staining controls were used to verify staining specificity. Histological staining was completed using staining kits and control slides while following the manufacturer’s instructions (Newcomer Supply, Middleton, WI, USA).

**Figure 2:**
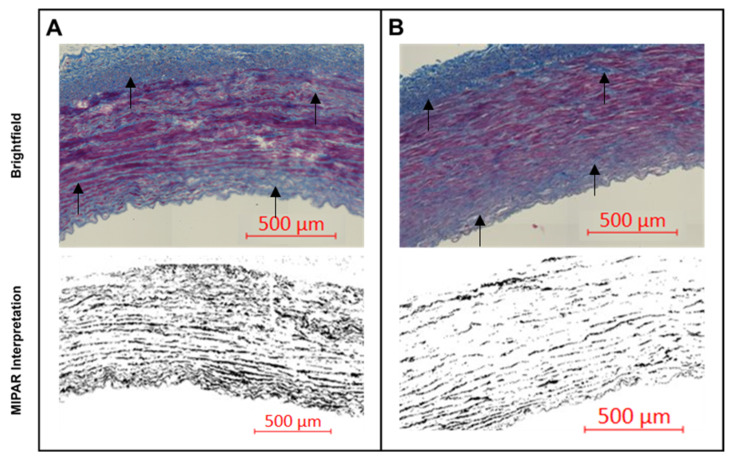
Representative images of the arterial wall from cross-sections stained for collagen using Trichrome staining following two hours of collagenase exposure. (**A**): Brightfield and MIPAR interpreted images of a POBA control artery. Black arrows on the brightfield images point to collagen fibers exhibiting degradation. (**B**): Brightfield and MIPAR interpreted images of a 10-8-10 Dimer-treated artery. Black arrows on the brightfield images point to collagen fibers showing less degradation positioned similarly as on panel (**A**).

Digital brightfield images of the stained slides were captured using a 10× objective on a Zeiss Axio Scan.Z1 located on the University of Utah campus at the Cell Imaging Core. Further morphometric analysis of the brightfield images was performed using MIPAR image analysis software (https://www.mipar.us accessed on 09 November 2023, v4.4.0, Columbus, OH, USA). MIPAR is an image analysis software that allows object-based image analysis (OBIA). OBIA allows for separation, classification, and quantification of microscopic features. Based on this concept, algorithms were developed for unbiased quantification of the amount of collagen fibers, elastin fibers, tunica media, and adventitial features. The less dense and less organized collagen corresponds with the presence of damaged collagen fibers. Intact collagen is organized in larger collagen bundles. The content algorithm uses feature colorings and shape algorithms to find the less dense and less organized collagen fragments by the Trichome staining, and as the signal gets translated into grayscale MIPAR interpretation of the images, the stronger signal reflects on larger amounts of degraded collagen (Figure 2). Intact collagen was used to calculate the mean fraction area of collagen present in the POBA control and 10-8-10 Dimer-treated arteries by taking the ratio of the area of intact collagen over the total analyzed artery area (see Section 3.3 Effect of Collagenase and Elastase Exposure).

**Figure 3:**
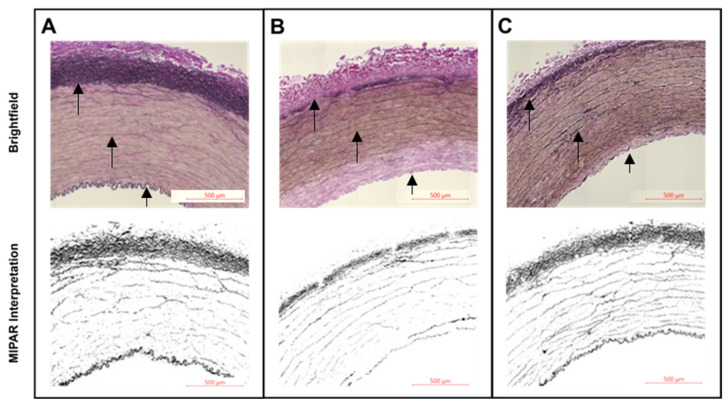
Representative images of the arterial wall from cross-sections stained for elastin using EVG staining following two hours of elastase exposure. (**A**): Brightfield and MIPAR interpreted images of untreated control artery. (**B**): Brightfield and MIPAR interpreted images of a POBA control artery. (**C**): Brightfield and MIPAR interpreted images of a 10-8-10 Dimer-treated artery. Across the three panels, black arrows point to the elastin fibers in three layers of the artery from outside to the lumen (top to bottom): adventitia, media, and internal elastic lamina (left to right for the three arrows). Panel (**B**) of the POBA control artery exhibits a reduction in the visibility of robust elastin fibers within the media after two hours of elastase exposure.

Morphometric analysis of the 10× digital brightfield images of EVG-stained elastin fibers was performed with MIPAR software. Specific algorithms were developed to discern and calculate the area of “black”-stained elastin fibers over the full area examined. The amount of elastin fibers was quantified as the mean area fraction percentage and compared between the POBA control and 10-8-10 Dimer-treated arteries (Figure 3 and Section 3.3 Effect of Collagenase and Elastase Exposure).

### 2.6. Statistical Analysis

Combinations of Microsoft Excel (Office16, Microsoft Office, Redmond, WA, USA), Minitab (21.4.2, Minitab, State College, PA, USA), and MATLAB (R2023a, The MathWorks Inc., Natick, MA, USA) were used for data processing and statistical analyses. Two-tail homoscedastic *T*-Tests were evaluated with a *p*-Value of <0.05 to claim statistical significance in Microsoft Excel. In addition to the statistical significance, 95% confidence interval error bars were included for each bar graph for illustrative purposes.

## 3. Results

### 3.1. Photochemical Dimerization of Tyr-Containing Pentapeptides

**Figure 4:**
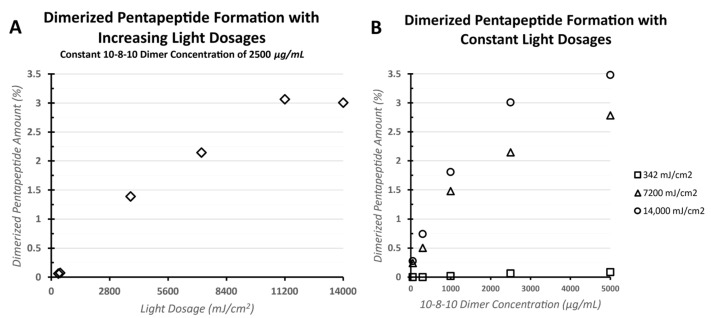
Dimerized pentapeptide formation measured by HPLC as a function of increasing light doses and 10-8-10 Dimer concentration. (**A**): Formation of dimerized pentapeptide with varying light dose. 10-8-10 Dimer (2500 µg/mL) was in excess of the model pentapeptide Ac-AKGYG-NH2 (650 µg/mL) and was kept constant. (**B**): Formation of dimerized pentapeptide at 3 constant light doses (14,000, 7200, and 342 mJ/cm^2^) with increasing 10-8-10 Dimer concentration.

To understand the photochemical mechanism of action of the 10-8-10 Dimer when exposed to light at 450 nm, a model system has been established to study the dimerization of the pentapeptide Ac-AKGYG-NH2, with dimerization occurring at the tyrosine (Tyr) residue [15,16]. Figure 4 demonstrates the light (A) and 10-8-10 Dimer (B) dependence of the dimerization reaction. In Figure 4A, the 10-8-10 Dimer concentration was held at 2500 µg/mL and the peptide held at 650 µg/mL. The amount of the dimerized pentapeptide formation increased with increasing light dose, with possible plateauing of this product formation at light doses ≥ 11,200 mJ/cm^2^. In Figure 4B, additional experiments were carried out at three different constant light doses, 342, 7200, and 14,000 mJ/cm^2^, respectively. The pentapeptide concentration again was held at 50 µg/mL (Figure 4B) and the 10-8-10 Dimer amount was varied at 50, 300, 1000, 2500, and 5000 µg/mL. Each light concentration showed an increase in the dimerized pentapeptide formation with increasing 10-8-10 Dimer concentration.

### 3.2. Cell Viability following Photochemical Treatment

The viability of HUVEC (mean ± SD) was 94.2 ± 21.4% with media control across all plates. The 10-8-10 Dimer appeared to have a negligible toxic effect on HUVEC cells in the absence of light exposure, with viability > 75% for almost all conditions, within the overall variability of the assay across the different conditions likely due to plating. The positive control, chlorpromazine, was effective, resulting in a dose-response decrease in cell viability. This was typically more severe in the presence of light, particularly in the longer post exposure times.

In the presence of 7 J/cm^2^ light dose, which represented the minimal light dose the vessel would be exposed to during in vivo treatment [9,18,19], cytotoxic effects were only observed with the highest concentration of the 10-8-10 Dimer (200 μg/mL) at 24, 48 and 72 h post exposure. The dose of 200 µg/mL is about 1000-fold above the dose of 10-8-10 Dimer which is delivered to the vascular tissues during in vivo or in vitro dosing with a 2 mg/mL 10-8-10 Dimer solution [17]. Increased cytotoxicity was observed with almost all exposure concentrations 48 h post treatment. However, the HUVEC cells seemed to recover over the next 24 h, and cytotoxicity was only observed 72 h post exposure at 200 μg/mL with the 7 J/cm^2^ light dose (Table 1).

In the presence of 14 J/cm^2^ light dose, which represents the maximal light dose the vessel would be exposed to during in vivo treatment, a somewhat increased cytotoxic effect was observed at 4, 24, 48 and 72 h post exposure to the increasing concentrations of 10-8-10 Dimer (Table 1). However, no cytotoxicity was detected at 12.5 and 25 µg/mL 10-8-10 Dimer exposure, which is 60- and 120-fold above the expected treatment concentration following in vitro and in vivo treatment [9,18,19]. Like the 7 J/cm^2^ light dose, the cells seemed to show better viability 72 h post exposure, with exception of the highest 10-8-10 Dimer dose (viability < 50% at 200 μg/mL).

The 28 J/cm^2^ light dose induced general toxicity at all concentrations and all post exposure times (Table 1) but was not toxic to the cells without the 10-8-10 Dimer present.

Overall, the treatment using 7 J/cm^2^ and 14 J/cm^2^ light doses and a 10-8-10 Dimer concentration not exceeding 25 µg/mL does not affect cell viability, providing a large, 60-120-fold, safety window for the treatment for both in vivo and in vitro applications.

### 3.3. Effect of Collagenase and Elastase Exposure

#### 3.3.1. Collagenase Exposure

**Figure 5:**
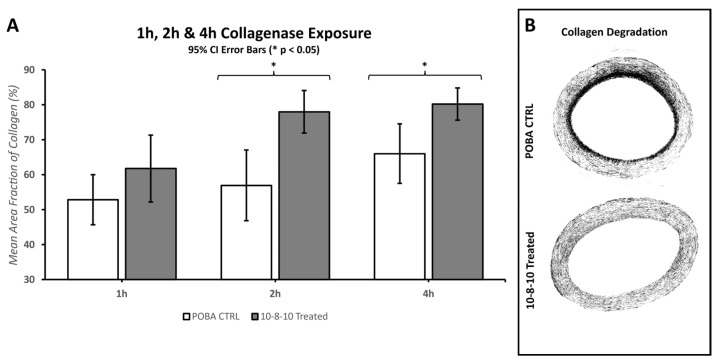
Amounts of 1 h, 2 h and 4 h exposure of POBA control and (N = 6, 7, 8, respectively, for each time point) porcine carotid arteries to collagenase. (**A**): The bar graphs show comparison between the amount of intact collagen remaining in the arteries after collagenase exposure of the POBA control (POBA CTRL) arteries (white bars) and the 10-8-10 Dimer-treated (10-8-10-treated) arteries (gray bars) at each time point. The 2 h and 4 h time points indicated statistical differences between POBA control and 10-8-10 Dimer-treated arteries with 95% confidence. The error bars shown are 95% confidence intervals. (**B**): Representative MIPAR generated interpretation of artery cross-sections from the 2 h samples created by the software. In this illustration, the more degraded collagen is depicted as black by the software algorithm. (* Two-tail homoscedastic *T*-Tests were evaluated with a *p*-Value of <0.05 to claim statistical significance).

The amount of collagen per cross-sectional area was determined using the MIPAR microstructure analytical software. The Trichrome-stained histological sections were subjected to a preprogrammed algorithm for unbiased detection of collagen fiber fragmentation. In Figure 5A, the vertical axis is a measurement of collagen with respect to the artery size, i.e., the percentage of the artery that is composed of intact collagen fibers. In comparison to POBA CTRL, 1 h incubation resulted in an 8% difference in the measured intact collagen, which was not statistically significant. However, more prolonged incubation, 2 h and 4 h, revealed the effect of the treatment by preserving 21% and 14% of the vascular collagen. The significant difference in the intact collagen amount in samples treated with the 10-8-10 Dimer prior to enzyme exposure compared to the POBA control samples indicates that the increase in the covalent bonds in the ECM by the photochemical treatment increased the resistance of the ECM to enzymatic destruction. Figure 5B depicts representative images of the MIPAR interpretation. The darker color in that case illustrates the more degraded, fragmented collagen (see methods).

#### 3.3.2. Elastase Exposure

**Figure 6:**
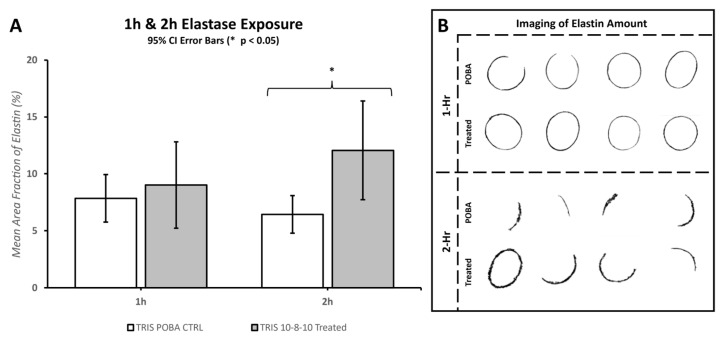
Amounts of 1 h and 2 h exposure of POBA control and 10-8-10 Dimer-treated (N = 6 and 9 for 1 h, and N = 5 and 9 for 2 h, respectively) porcine carotid arteries to elastase. (**A**): The bar graphs show comparisons between the amount of elastin remaining in the arteries after elastase exposure of the POBA control (POBA CTRL) arteries (white bars) and the 10-8-10 Dimer-treated (10-8-10-treated) arteries (light gray bars) at the two depicted time points. The vertical axis is a measurement of elastin with respect to the artery size, i.e., the percentage of the artery that is composed of elastin fibers. The 2 h time point indicated statistical differences between the POBA control and the 10-8-10 Dimer-treated arteries with 95% confidence. The error bars shown are 95% confidence intervals. (**B**): MIPAR interpretation of various POBA control (POBA) and 10-8-10 Dimer-treated artery cross-sections used for the quantitative image analysis by the software, one hour and two hours following elastase exposure. Intact elastin is depicted as black by the software algorithm. (* Two-tail homoscedastic *T*-Tests were evaluated with a *p*-Value of <0.05 to claim statistical significance).

Similar to the collagenase treatment, the 10-8-10 Dimer-treated or POBA control arteries were cut into smaller segments for the incubation with elastase. The EVG-stained histological sections (see Figure 3) were subjected to a preprogrammed algorithm for unbiased determination and quantitation of the remaining intact elastin in the vessel wall following 1 and 2 h enzymatic digestion of the arterial segments by elastase. Figure 6 shows the remaining elastin in the vessel wall. Significant protection of elastin in the 10-8-10 Dimer-treated arteries is apparent after 2 h digestion with elastin (Figure 6A). Figure 6B illustrates the cross-sectional distribution of remaining intact elastin, as determined by the software in the adventitial layer of the artery. The images depict the effect of elastase degrading the vascular elastin through time in enzyme solution. The amount of enzymatic digestion is reduced in samples that received the photochemical treatment.

## 4. Discussion

This study examined the molecular-, cellular-, and tissue-level effects of the innovative photochemical treatment, known as Natural Vascular Scaffolding, aimed at facilitating vascular remodeling upon treatment. Our results reveal that the 10-8-10 Dimer and light doses utilized in in vivo models [9,17] is capable of dimerizing Tyr-containing synthetic peptides and do not adversely impact cellular viability. Instead, they safeguard the ECM components within the vessel wall from enzymatic degradation. This ECM protection forms the foundation for the long-term remodeling benefits of this treatment, as previously demonstrated in in vivo animal models [9,17].

The pathogenesis of many cardiovascular diseases has been associated with loss of organization and function of the ECM. The key findings in our study revealed that the elastin and collagen constituents of the vessel wall exhibit diminished enzymatic degradation following Natural Vascular Scaffolding treatment compared to vascular tissues stretched with plain old balloon angioplasty (POBA). The ECM’s capacity for simultaneous stability and adaptability within this protein fiber mesh is essential for its role as a scaffold. Preserving the ECM framework is pivotal for enabling a controlled adaptive response orchestrated by vascular cells of the vessel wall in response to changes in hemodynamic and metabolic conditions [5].

The dynamic nature of the ECM is largely influenced by the degree of crosslinking among the proteins forming the matrix. Achieving optimal stabilization of the native ECM for a wide range of biological applications is best accomplished through photochemical crosslinking [14]. Photochemical crosslinking treatments with demonstrated or potential utility for medical applications include photodynamic therapy (PDT), photochemical tissue passivation (PTP), and natural vascular scaffolding (NVS). Despite sharing the same photochemical principle, these applications differ in the application of light, wavelength and irradiance delivered, the type of photoactive molecules, and the duration of exposure in the proximity of the target tissue. In the context of PDT, the activation of light initiates the formation of intracellular reactive oxygen species (ROS). The surplus ROS trigger apoptotic pathways, inducing cell death, a technique exploited in cancer therapy [18]. PDT also proves effective as an antimicrobial treatment for oral infections [19]. PTP is generally characterized as a photochemical treatment resulting in reduced tissue inflammation and enhanced stiffening of the treated tissues, such as the cornea. Riboflavin and Rose Bengal are the two most studied photoactive molecules used as PTPs for therapeutic use. In PTP, a sterile solution containing the photoactive molecule is applied to the target tissue, and then the tissue is exposed to light with wavelength as maximum absorption (550 nm for Rose Bengal and 365 nm for riboflavin). The duration of light exposure is 15 min for Rose Bengal [20,21] and 30 min for riboflavin [22] to create the desired cross-linking in the treatment area. Despite these differences, their photophysical properties are similar [23].

The 10-8-10 Dimer has an absorption maximum at 450 nm. Upon exposure to 450 nm light, the photosensitizer 10-8-10 Dimer is photoexcited to a triplet excited state. That triplet excited state can convert triplet oxygen to singlet oxygen, which would crosslink amino acids via H-atom extraction, or the triplet excited 10-8-10 Dimer can convert to an intramolecular charge transfer (ICT state) which would directly oxidize the amino acids followed by amino acid crosslinking [15]. When two such amino acid sites are in proximity, they can form covalent bonds, thus crosslinking the protein molecules. This process does not exclusively target specific amino acids but it primarily involves amino acids that are prone to oxidation.

We used a model peptide with Tyr residues in our experiments [15,16]. The conversion of Tyr residues into diTyr-linked products through oxidation is a well-studied process. The covalent o,o’-Tyr-Tyr cross-link is easily identifiable and quantifiable. We have previously optimized the conditions to measure the Tyr crosslinking formation by 450 nm light-activated 10-8-10 Dimer [16]. In the present study, we show that this crosslinking effect can be achieved at 10-8-10 Dimer concentrations, which can be delivered to the vessel wall either in vitro or in vivo [17].

Due to the lack of selectivity of photoinduced oxidative processes and the wide use of PDT [18,19], we have evaluated the potential cytotoxicity of the treatment using endothelial cells in culture. Cultured endothelial cells serve as valuable models for investigating the impact of drug treatments on reendothelialization. These actively proliferating endothelial cells in culture effectively mimic the cellular response to acute vascular injury resulting from endovascular procedures or surgical manipulation of vascular segments [24]. In a previous study, light-activated 10-8-10 Dimer treatment, conducted under conditions matching those used in the in vivo rat arteriovenous fistula model, induced minimal cell death through apoptosis [9]. In this study, investigations were expanded to include a wide range of 10-8-10 Dimer concentrations and evaluated light exposure levels within the range expected during in vivo treatment (ranging between 7 and 14 J/cm^2^) while also doubling the light dose beyond these levels (28 J/cm^2^). The findings presented in Table 1 provide robust evidence of the treatment’s cellular safety across this wide range. This underscores the technology’s relative selectivity compared to other photochemical methods like photochemical tissue passivation (PTP) and PDT, with the latter primarily aiming to induce cell death. The absence of cell death was also evident in previous contractility studies involving freshly isolated human popliteal arteries subjected to the same photochemical treatment using the 10-8-10 Dimer and 450 nm light, mirroring the conditions applied to porcine carotid arteries in the current experiments [25]. These studies indicated that the contractile responsiveness of the vascular smooth muscle to vasoactive pharmacological stimulation is retained following the photochemical treatment [25].

In this study, the application of balloon stretch during treatment played a crucial role in observing the degradation of both collagen and elastin within hours of enzyme exposure. Despite the mechanical stretch aiding in enzymatic degradation, there was no significant difference between the photochemically treated segments and the POBA control in the first hour of enzyme treatment. A significant difference emerged by the second hour of the enzyme treatment. However, the extent of tissue damage caused by the elastase enzyme treatment leveled out by the fourth hour, making it difficult to reliably measure any difference between the POBA control and photochemically treated tissues. Consequently, the 2 h incubation period was deemed the most optimal for investigating the impact of the 10-8-10 Dimer and blue light treatment in the case of elastin enzyme treatment represented in Figure 6. To objectively quantify changes in fiber integrity, we employed the microstructure analysis software MIPAR (https://www.mipar.us/, accessed on 09 November 2023). Specific algorithms were developed using Masson Trichrome- and EVG-stained histological sections of the arteries to detect variations in collagen and elastin fiber integrity between the POBA control and 10-8-10 Dimer- and 450 nm light-treated vascular sections, respectively. Our results demonstrate a significant protection of both collagen and elastin fibers from enzymatic destruction, which supports the notion of its impact on vascular remodeling. It is important to note that the analysis’ impartial nature has limitations. The quality of the processed tissue, as well as the histological sections and staining quality, significantly influence the analysis. Hence, we took special care during tissue processing to ensure the production of high-quality histological sections.

Vascular injury as a result of endovascular balloon stretches leads to ECM fragmentation and inflammation [26,27]. Inflammatory processes create a cascade of events systemically as well as in the vascular wall, which further activate the original scaffold destruction and the lost directionality of cell proliferation results in stenosis. Our previous studies have pointed to the potential anti-inflammatory effect by the photochemical treatment using the 10-8-10 Dimer and 450 nm light in in vitro studies using freshly isolated human popliteal arteries in organ culture or in vivo in the rat arteriovenous fistula studies [9,25]. Due to the nature of those experiments, the direct effect of the photochemical treatment on the ECM proteins could not be separated from its possible indirect effect on the inflammatory process itself or its potential inhibitory effect on the activity of the endogenously present matrix-degrading proteins. The current study allows us to further elucidate the mechanism of this protection, which points to the direct effect of the photochemical treatment on the ECM scaffold. In these experiments, freshly isolated carotid arteries were used in vitro from young animals without sign of vascular disease. The photochemical treatment preceded the exposure to enzymatic digestion, ruling out the possibility that the enzyme would have been deactivated by the photochemical treatment. Based on these experiments, the protection of the ECM fibers from enzymatic degradation is attributed to the covalent crosslinks as a result of the photochemical treatment.

Differences in cell viability and phenotype, as well as fluctuations in the deposition and degradation of the individual fiber components within the extracellular matrix (ECM), are interlinked processes. The ECM scaffold serves as the microenvironment for cellular components, where the regulation of proliferation and inflammation takes place. A successful cellular response during adaptive vascular remodeling hinges on the viability of vascular cells and their phenotypic switch, which are both significantly influenced by their ongoing interactions with the vascular ECM [1,2,3]. It is evident that any shifts in the ECM’s integrity will impact the active proliferative remodeling process. In most instances, these environmental cues are detected by cell surface receptors. These receptors trigger chemical or mechanical signal transduction, leading to modifications in gene expression and subsequent gene product changes [28]. A significant number of these changes in gene expression influence the composition and arrangement of the ECM, ultimately directing alterations in the tissue’s structure and biomechanical properties [29]. These adjustments play a fundamental role in shaping the overall functionality of the tissue.

The substantial loss of elastin content, accompanied by the fragmentation of elastic laminae and fibers, is a well-documented feature of aneurysms, a pathological condition marked by stiffened and dilated arterial walls. In line with our current results regarding the preservation of vascular elastin by the photochemical treatment, our prior investigations utilizing the mouse elastase model of aortic abdominal aneurysms revealed a delayed onset of aneurysm development and the maintenance of smooth muscle cell content in aortas that had previously undergone treatment with 10-8-10 Dimer and blue light [30]. This underscores the potential therapeutic value of the photochemical treatment in managing maladaptive remodeling conditions.

An illustrative case for the necessity of adaptive, physiological remodeling is the maturation of AVFs, where the quality and integrity of the ECM plays a pivotal role [8]. Excessive fibrotic remodeling in the vein following AVF creation can impede outward remodeling and has been recognized as a major contributor to the risk of maturation failure [8]. In our prior research using a rat model of AVF surgery, we demonstrated substantial benefits from our photochemical treatment administered at the time of fistula creation. This treatment led to the outward remodeling of the venous wall within four weeks after treatment, coinciding with the arterialization process, while preserving fistula patency [9]. More recently, we conducted a study in sheep, administering the photochemical treatment via a specially designed intravascular device for AVF creation. In these studies, the veins in treated sheep exhibited significantly larger lumens and thicker vein walls within seven days following treatment. This result was accomplished by using our photochemical treatment while stretching the veins with a coated balloon device. The balloon was coated with the 10-8-10 Dimer and the blue light was administered through a light fiber positioned inside the balloon. This process, in turn, facilitated adaptive cell proliferation and resulted in accelerated maturation of the fistula veins.

## 5. Conclusions

Our findings corroborate prior in vivo observations involving the Natural Vascular Scaffolding technology, indicating that the photochemical treatment with the 10-8-10 Dimer and blue light safeguards the organization and stability of the ECM. Importantly, this effect is achieved at treatment doses that do not harm cellular integrity, thereby permitting ongoing adaptation and remodeling of the treated vascular wall in response to hemodynamic demands. The technology’s non-detrimental effects on cellular viability underscore its safety and suitability for clinical use. The Natural Vascular Scaffolding technology holds significant promise for clinical applications in the field of vascular medicine.

## Figures and Tables

**Table 1:** HUVEC viability over 4, 24, 48 and 72 h post exposure incubation following in vitro treatment with different concentrations of the 10-8-10 Dimer and 450 nm light. Cell viability is expressed as an average of the quadruplicate wells (n = 4) for each treatment as percent (%) of the control cells’ viability. Green color represents >75% viability within the error margin of the study, orange color represents >75–<50% acceptable viability, dark pink color represents <50% viability indicating cytotoxic effect. The 10-8-10 Dimer concentration is listed on the left-hand side of the table (drug dose) and the treatment was carried out at three different light dose exposures. The doses 7 J/cm^2^ and 14 J/cm^2^ represent the minimal and maximal light during in vivo and in vitro vascular treatment. Treatment is safe for the cellular elements of the vessel wall up to a 60-120-fold higher 10-8-10 Dimer concentration up to 14 J/cm^2^ light exposure.

10-8-10 Dimer Dose	7 J/cm^2^				14 J/cm^2^				28 J/cm^2^			
	4 h	24 h	48 h	72 h	4 h	24 h	48 h	72 h	4 h	24 h	48 h	72 h
12.5 µg/mL	93	97	70	120	98	75	63	119	58	42	33	18
25 µg/mL	91	99	68	108	94	76	66	96	56	33	27	17
50 µg/mL	99	107	64	102	99	75	63	59	57	27	26	13
100 µg/mL	100	142	110	139	103	97	51	61	61	31	30	18
200 µg/mL	114	71	50	41	78	19	21	13	47	24	22	9

## Data Availability

The data presented in this study are available on request from the corresponding author (K.K.).
